# Evaluating the Methodological Quality of Artificial Intelligence–Assisted Systematic Reviews: Protocol for a Mixed Methods Meta-Research Study

**DOI:** 10.2196/90588

**Published:** 2026-05-14

**Authors:** Mohammad Jay, Mary Morgan, Sharon Elizabeth Straus, Emma Wilson, Rinku Sutradhar, Catherine Yu, Lesley Gotlib Conn, Christoffer Dharma, Lorraine Lipscombe, Antoine Eskander

**Affiliations:** 1 Department of Medicine, Division of Endocrinology, University of Toronto, 2075 Bayview Avenue, Toronto, ON, M4N 3M5, Canada, 1 416-480-6705, 1 416-480-5761; 2 Institute of Health Policy, Management and Evaluation (IHPME), Dalla Lana School of Public Health, University of Toronto, Toronto, ON, Canada; 3 Faculty of Medicine, University of Toronto, Toronto, ON, Canada; 4 Department of Medicine, Division of Geriatric Medicine, University of Toronto, Toronto, ON, Canada; 5 Knowledge Translation Program, Li Ka Shing Knowledge Institute, University of Toronto, Toronto, ON, Canada; 6 Library Services, Sunnybrook Health Science Centre, Toronto, ON, Canada; 7 Institute for Clinical Evaluative Sciences, Toronto, ON, Canada; 8 Division of Biostatistics, Dalla Lana School of Public Health, University of Toronto, Toronto, ON, Canada; 9 Department of Medicine, Women's College Hospital, Toronto, ON, Canada; 10 Department of Otolaryngology–Head and Neck Surgery, Sunnybrook Health Sciences Centre, University of Toronto, Toronto, ON, Canada

**Keywords:** systematic review, meta-research, artificial intelligence, large language models, AMSTAR-2, PRISMA 2020, transparency, reproducibility, evidence synthesis

## Abstract

**Background:**

Artificial intelligence (AI), including large language models (LLMs), is increasingly integrated into systematic review (SR) workflows. AI tools may accelerate searching, screening, data extraction, and reporting, but their effects on methodological quality, reporting completeness, transparency, and reproducibility remain uncertain. Existing evaluations largely examine isolated tasks, and inconsistent disclosure of AI use limits reproducibility and oversight.

**Objective:**

This 4-phase mixed methods meta-research study will (1) compare the methodological quality of AI-assisted versus traditional SRs; (2) refine, finalize, and apply a preliminary AI Transparency and Disclosure Index (AITDI); (3) evaluate reproducibility by comparing outputs across repeated runs of the same AI model, across different AI models, and between AI models and human reviewers at multiple SR stages; and (4) explore knowledge user perspectives on rigor, transparency, and trust in AI-assisted SRs.

**Methods:**

We will conduct a matched cohort analysis of SRs published from 2023 to 2025 in biomedical journals. Each AI-assisted SR will be matched 1:2 with traditional SRs by publication year, clinical domain, review type, and meta-analysis status. Two independent reviewers will apply A Measurement Tool to Assess Systematic Reviews, version 2 (AMSTAR 2; methodological quality), PRISMA (Preferred Reporting Items for Systematic Reviews and Meta-Analyses) 2020 (reporting completeness), and, when applicable, Risk of Bias in SRs (ROBIS; risk-of-bias rigor). A preliminary AITDI will be refined and then applied to all AI-assisted SRs. Reproducibility will be assessed using SR-derived task sets to compare outputs across repeated runs of the same model, across different models, and between AI and human reviewers at key SR stages. Semistructured interviews with authors, editors, clinicians, policymakers, and patient partners will be analyzed using reflexive thematic analysis.

**Results:**

As of December 2025, the study has been preregistered on the Open Science Framework (OSF; DOI: 10.17605/OSF.IO/Q5JRW), the search strategy has been finalized, and title/abstract screening has begun. Data extraction is planned for March-May 2026, followed by AITDI refinement and reproducibility testing from May 2026 to October 2026. Qualitative interviews are anticipated from October 2026 to February 2027, with final analyses by April 2027 and dissemination planned for mid-2027.

**Conclusions:**

This study will provide one of the first empirical comparisons of methodological quality, transparency, and reproducibility of AI-assisted versus traditional SRs in the LLM era. Findings will inform expectations for responsible AI integration and support refinement of reporting and methodological best practices, including future development of AI-specific reporting and appraisal extensions (eg, PRISMA-LLM [Preferred Reporting Items for Systematic Reviews and Meta-Analyses–large language model] and AMSTAR-LLM [A Measurement Tool to Assess Systematic Reviews-large language model]).

## Introduction

### Background

Systematic reviews (SRs) and meta-analyses synthesize evidence using transparent, reproducible methods and inform guidelines and policy [1]. However, traditional SR workflows are resource-intensive and often outdated by publication. Throughout this manuscript, we use the term “traditional systematic reviews” to refer to human-driven systematic reviews (ie, reviews conducted without reported artificial intelligence [AI] assistance). Interest in AI, particularly large language models (LLMs), reflects the need to accelerate these processes [2]. As AI tools are applied to screening, data extraction, and drafting, concerns arise about whether AI-assisted SRs preserve methodological rigor and appropriate human oversight.

### AI in SRs

AI tools (eg, machine learning classifiers, natural language processing, and LLMs) are increasingly used for searching, screening prioritization, data extraction, risk-of-bias assessment, and reporting. Platforms such as Abstrackr (Brown University) [3], Rayyan (Rayyan Systems Inc) [4], DistillerSR (Evidence Partners Inc) [5], and RobotReviewer (King’s College London) [6] integrate automated features to streamline workflows. While AI may improve efficiency, concerns persist regarding transparency, reproducibility, and errors that may influence clinical or policy decisions. Evaluations must therefore consider the quality of completed SR rather than isolated tasks.

### Existing Evidence

Evidence to date is limited and mixed. Prior studies suggest potential efficiency gains with a broadly similar tool, A Measurement Tool to Assess Systematic Reviews version 2 (AMSTAR 2), scores in selected settings [7] and variable agreement for specific appraisal tasks [8,9]. Others caution that errors, inconsistent outputs, and poor disclosure may compromise trust and reproducibility [5,10]. Overall, the literature largely evaluates discrete tasks or narrow clinical areas, with limited assessment of the methodological and reporting quality of fully published AI-assisted SRs, AI transparency, cross-run/model reproducibility, or knowledge user expectations.

### Evidence Gap in the LLM Era

LLM use in SR workflows has expanded rapidly since 2023; yet, reporting of AI involvement is inconsistent. Existing appraisal (eg, AMSTAR 2 [11] and the Risk of Bias in SRs [ROBIS] [12]) and reporting tools (eg, the PRISMA [Preferred Reporting Items for Systematic Reviews and Meta-Analyses] 2020 [13]) assess general methodological rigor and reporting completeness (eg, protocol registration, search comprehensiveness, duplicate processes, and risk-of-bias appraisal), but do not capture AI-specific details such as tool identity, model version, prompting, stage of use, or human oversight. To address this gap, we developed a preliminary AI Transparency and Disclosure Index (AITDI; Table 1).

**Table 1.** Preliminary artificial intelligence transparency and disclosure index (AITDI): domains and minimal reporting requirements.

Domain	Description	Minimal reporting requirement
Tool identity	Identifies the AI^a^ system used (eg, screening classifier, LLM^b^, and extraction tool).	State the specific tool or model used (eg, ChatGPT [OpenAI] and Rayyan [Rayyan Systems Inc] classifier).
Model version or date	AI models update frequently; versioning is essential for reproducibility.	Provide the version number, release date, or retrieval date.
Stage of use	Specifies which systematic review stages involved AI.	List all workflow stages where AI contributed (eg, screening, extraction, appraisal, synthesis, and drafting).
Parameter settings or prompts	Prompts and settings (eg, temperature) influence outputs.	Report prompts, parameters, thresholds, or configurations used.
Human oversight	Describes reviewer verification, correction, and judgment.	Specify the oversight structure and degree of human verification.
Data governance and ethics	Notes privacy, confidentiality, and security issues relevant to AI use.	Describe data handling safeguards and ethical considerations.
Recommended (nonscored): AI-related limitations	AI risks such as hallucinations, instability, or model drift affect interpretability but do not distinguish reviews.	Provide a brief statement acknowledging AI-specific limitations (not scored).

a AI: artificial intelligence.

b LLM: large language model.

### Purpose and Significance

This study responds to emerging use of AI in SR workflows by providing a comprehensive evaluation of AI-assisted SRs at the level of completed reviews. By integrating assessment of methodological quality, reporting completeness, transparency, reproducibility, and knowledge-user perspectives, this work moves beyond task-level evaluations to address how AI affects review quality of SRs in practice. The findings will inform best practices for responsible AI integration and support future development of AI-specific reporting and appraisal extensions.

### Objectives

**Figure 1.**
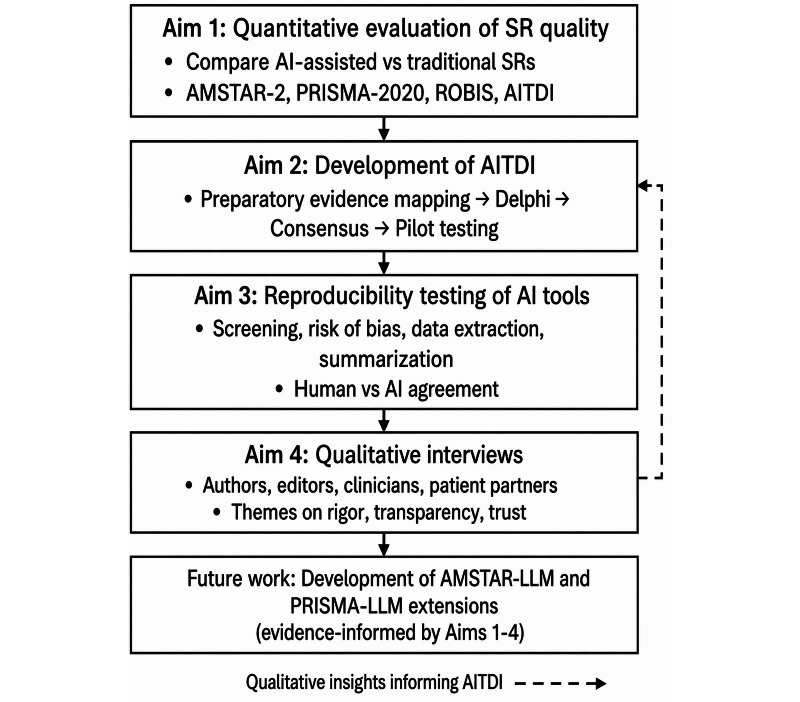
Study design schematic for aims 1‐4 and planned future tool development. AI: artificial intelligence; AITDI: Artificial Intelligence Transparency and Disclosure Index; AMSTAR 2: A Measurement Tool to Assess Systematic Reviews version 2; LLM: large language model; PRISMA 2020: Preferred Reporting Items for Systematic Reviews and Meta-Analyses 2020; PRISMA-LLM: Preferred Reporting Items for Systematic Reviews and Meta-Analyses–large language model; ROBIS: Risk of Bias in Systematic Reviews; SR: systematic review.

This 4-phase mixed methods meta-research study aims to (1) compare the methodological quality of AI-assisted versus traditional SRs; (2) refine, validate, and apply a preliminary AITDI to evaluate AI-related reporting; (3) assess the reproducibility of AI-assisted SR processes across repeated runs, different models, and human comparators; and (4) explore knowledge user perspectives on rigor, transparency, oversight, and trust in AI-assisted SRs. Figure 1 provides a schematic overview of the four study aims and how they interrelate within the mixed methods design.

## Methods

### Study Design

**Figure 2.**
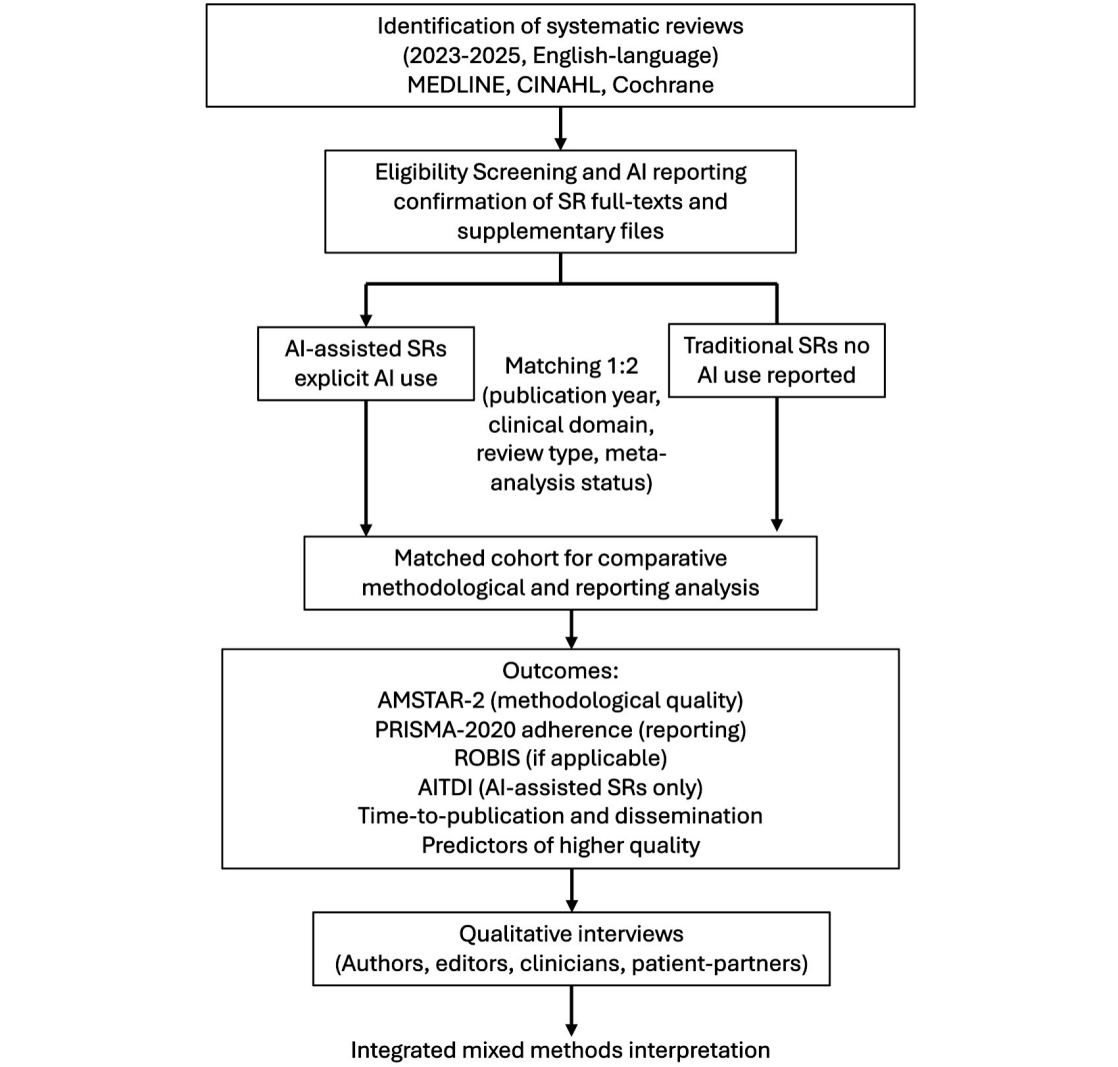
Workflow for aim 1 and integration with aim 4. Overview of the processes for identifying, matching, extracting, and appraising systematic reviews, followed by comparative analyses and integration with qualitative themes. AI: artificial intelligence; AITDI: AI Transparency and Disclosure Index; AMSTAR 2: A Measurement Tool to Assess Systematic Reviews version 2; PRISMA 2020: Preferred Reporting Items for Systematic Reviews and Meta-Analyses 2020; ROBIS: Risk of Bias in Systematic Reviews; SR: systematic review.

This is a 4-aim mixed methods meta-research study. Aim 1 is a matched-cohort comparison of AI-assisted and traditional SRs published between 2023 and 2025 (the LLM era [14]). Aim 2 refines and validates the AITDI, following best practices for reporting guideline development (Moher et al [15]). Aim 3 evaluates the reproducibility of AI outputs using SR-derived task sets. Aim 4 uses qualitative interviews to explore knowledge-user perspectives. Figure 2 summarizes the workflow for Aim 1 and integration with Aim 4.

### Aim 1: Comparative Quality Study

#### Eligibility Criteria

Eligible records will consist of peer-reviewed published articles that self-identify as SRs, with or without meta-analysis, written in English, and indexed in MEDLINE, CINAHL, or the Cochrane Database of Systematic Reviews. SRs must address health-related questions (human health outcomes, public health, health care delivery, or health-system decision-making). AI-assisted SRs must be published between January 1, 2023, and December 31, 2025. To support time-proximal matching (±3 months; see “Matching Strategy”), traditional (non–AI-assisted) comparator SRs may be published between October 1, 2022, and March 31, 2026, provided they meet all other eligibility criteria. We will exclude scoping reviews, rapid reviews, evidence maps, narrative reviews, umbrella or overview reviews, as well as protocols, conference abstracts, letters, commentaries, editorials, preprints, and reviews focused exclusively on preclinical or animal research. Reviews lacking full-text availability after reasonable efforts to obtain the article (including contacting the corresponding author when appropriate) or a reproducible search strategy will also be excluded. Title/abstract screening will confirm SR status, health relevance, and publication characteristics. Classification of AI will occur at full-text review based on explicit reporting in the manuscript or supplementary materials. Table 2 summarizes the population, intervention, comparator, and outcome (PICO) eligibility criteria for included SRs.

**Table 2.** Population, intervention, comparator, and outcome (PICO) eligibility criteria for included systematic reviews

Domain	Eligibility criteria
Population	Systematic reviews focused on human health, clinical medicine, public health, or health care–related topics. No restrictions on patient age, condition, or setting.
Intervention / Exposure	Explicit use of artificial intelligence (AI)^a^, machine learning, natural language processing, large language models (LLMs)^b^, or automation tools at any stage of the systematic review workflow (eg, searching, screening, data extraction, risk-of-bias assessment, synthesis, or drafting).
Comparator	Traditional (non–AI-assisted) systematic reviews matched 1:2 to AI-assisted SRs^c^ by publication year, review type, clinical domain, and presence/absence of a meta-analysis.
Outcomes	Methodological quality (AMSTAR 2)^d^, reporting completeness (PRISMA 2020)^e^, risk-of-bias rigor (ROBIS)^f^, AI transparency (AITDI^g^; AI-assisted SRs only), and secondary outcomes including timeliness to publication, dissemination metrics, and workload indicators (when available).

a AI: artificial intelligence.

b LLM: large language model.

c SR: systematic review.

d AMSTAR 2: A Measurement Tool to Assess Systematic Reviews version 2.

e PRISMA 2020: Preferred Reporting Items for Systematic Reviews and Meta-Analyses 2020.

f ROBIS: Risk of Bias in Systematic Reviews.

g AITDI: AI Transparency and Disclosure Index.

#### Exposure and Comparator Definitions

The exposure is author-reported AI assistance, operationalized as explicit documentation of AI, machine learning, natural language processing, or LLMs at any stage of the SR workflow, including searching, screening or prioritization, data extraction, risk-of-bias assessment, evidence synthesis (qualitative/narrative or quantitative/meta-analytic), or manuscript drafting. Evidence of AI use may appear in the main text, supplementary materials, acknowledgments, or dedicated disclosure statements. The comparator will include SRs meeting all other eligibility criteria with no reported AI use in the available published materials. Comparator publication dates will align with the parameters specified in “Eligibility Criteria” to permit time-proximal matching. Conventional software without embedded AI functionality (eg, Covidence [Veritas Health Innovation Ltd], RevMan [Review Manager], and Excel [Microsoft Corp]) will be classified as non–AI-assisted.

AI involvement will be classified by stage and depth of integration rather than treated as a binary. Core methodological uses will include AI contributing directly to evidence identification, selection, appraisal, or synthesis (eg, searching, screening, data extraction, risk-of-bias assessment, or synthesis), whereas supporting use will include drafting or language editing only. Because exposure classification depends on explicit reporting, some comparator reviews may have involved unreported AI use. If such misclassification is nondifferential with respect to methodological quality, it would be expected to attenuate observed between-group differences toward the null. Accordingly, Aim 1 should be interpreted as primarily comparing reviews with reported AI use versus reviews with no reported AI use.

### Matching Strategy

#### Rationale for Matching

A matched cohort design minimizes temporal and topic-related confounding that could independently influence review quality. Matching ensures that AI-assisted and traditional SRs are compared within the same publication period and clinical context, reducing bias from secular trends in SR methodology, evolving journal standards, or variation across clinical domains.

#### Matching Factors

Each AI-assisted SR will be matched to two traditional SRs based on four prespecified characteristics: (1) publication year (within ±3 mo) to control for rapidly evolving norms in the LLM era; (2) clinical domain, operationalized using major MeSH (Medical Subject Headings) terms or journal specialty classification; (3) review type (standard or living review); and (4) meta-analysis status (presence vs absence of quantitative synthesis). When multiple eligible comparators exist, two will be selected using a reproducible randomization procedure.

#### Why a 1:2 Ratio

A 1:2 matching ratio increases statistical power and precision relative to 1:1 matching while maintaining feasibility given the expected smaller pool of AI-assisted SRs. This ratio balances efficiency with practicality and follows best practices in meta-epidemiologic design [16].

### Outcomes

#### Quality Assessment Instruments

Methodological quality will be assessed using AMSTAR 2, a validated 16-item appraisal tool covering key domains including protocol registration, comprehensive searching, duplicate processes, risk-of-bias assessment, and appropriateness of synthesis methods [17]. Reporting completeness will be evaluated using the 27-item PRISMA 2020 checklist [13] and risk of bias in review methods using ROBIS [12], which assesses eligibility criteria, study selection, data collection and appraisal, and synthesis and interpretation. Transparency of AI use will be assessed using the AITDI, developed for this study, which includes 6 domains, namely tool identity, stage of use, model version, prompting or configuration details, human verification, and data-privacy or ethical statements, along with a recommended nonscored domain addressing AI-related limitations (eg, hallucinations or model drift). The development of the AITDI as a preliminary framework is described under Aim 2, and the index domains are summarized in Table 1.

#### The Primary Outcome

The primary outcome will be methodological quality, measured using the AMSTAR 2 tool and expressed as a modified 0‐13 item-level adherence score. In this meta-research study, this operational summary measure was selected because the primary estimand is the average difference in methodological adherence between groups of systematic reviews, rather than the appraisal of individual reviews for decision-making purposes. A continuous item-level score allows estimation of absolute group-level differences and supports a prespecified noninferiority framework. Relative to coarse ordinal confidence categories, a continuous item-level adherence measure preserves more information and allows more sensitive detection of average between-group differences in methodological adherence. We recognize that AMSTAR 2 was originally designed to support appraisal through critical domains and overall confidence ratings. However, those overall confidence categories are intentionally coarse and nonlinear and are designed to reflect critical weaknesses in individual reviews rather than average between-group differences in methodological adherence in a matched comparative meta-research design. Accordingly, we will use a modified item-level adherence score as the primary analytic outcome while interpreting results in conjunction with AMSTAR 2 overall confidence ratings and prespecified critical domains. Although AMSTAR 2 was not developed as a formal additive scale, similar item-level summary approaches have been used in prior meta-research to facilitate group-level comparisons [7] and should be interpreted cautiously alongside standard AMSTAR 2 confidence ratings. Consistent with the structure of the included review sample, items that are conditional on quantitative synthesis (AMSTAR 2 items 11, 12, and 15) will be excluded from the summary measure, yielding a maximum possible score of 13. These items will still be extracted for reviews that include meta-analysis and will be summarized descriptively and in subgroup analyses restricted to meta-analytic reviews. This modified score is intended as a group-level comparative metric rather than a replacement for standard AMSTAR 2 interpretation at the level of individual reviews. Critical AMSTAR 2 domains (eg, protocol registration, duplicate processes, and comprehensiveness of the search) will also be summarized descriptively.

#### Secondary Outcomes

**Figure 3.**
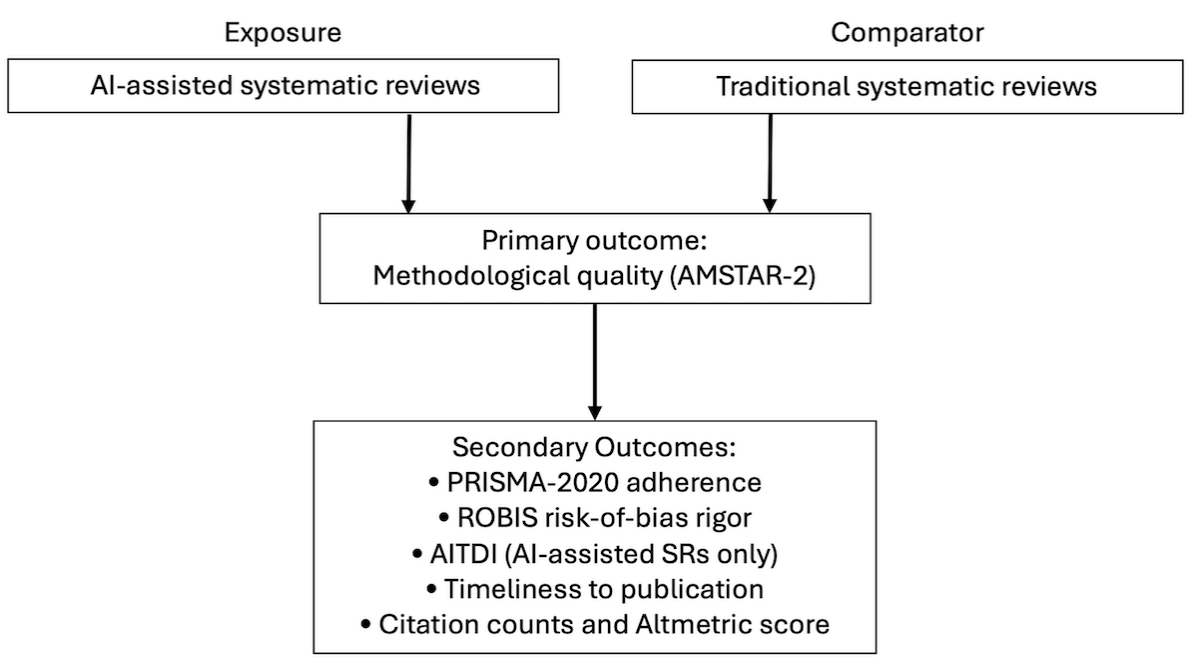
Exposure-outcome schema for Aim 1. The exposure is artificial intelligence (AI)–assisted systematic reviews compared to traditional systematic reviews. The primary outcome is methodological quality (AMSTAR 2). Secondary outcomes include reporting quality (PRISMA 2020), risk-of-bias rigor (ROBIS), AI transparency (AITDI), timeliness to publication, and dissemination metrics. AI: artificial intelligence; AITDI: AI Transparency and Disclosure Index; AMSTAR 2: A Measurement Tool to Assess Systematic Reviews version 2; PRISMA: Preferred Reporting Items for Systematic Reviews and Meta-Analyses; ROBIS: Risk of Bias in Systematic Reviews; SR: systematic review.

This will include overall reporting quality (percentage adherence to PRISMA 2020), risk of bias rigor (domain-level and overall ROBIS judgments, when applicable), and transparency of AI use measured using the preliminary AITDI score (an additive item-level score reflecting completeness of AI-use disclosure across 6 domains; applied provisionally in Aim 1, with full refinement and validation in Aim 2). Exploratory outcomes will include timeliness, defined as the interval between protocol registration (or earliest submission) and publication, and dissemination metrics such as 12-month citation counts and Altmetric Attention Scores. Exploratory analyses will also examine whether characteristics such as review type (standard or living), inclusion of meta-analysis, journal tier, team size, and clinical domain are associated with higher methodological or reporting quality. Figure 3 presents the exposure-outcome schema for the comparative analyses. Multimedia Appendix 1 provides a detailed summary of all primary and secondary outcomes and their corresponding quality assessment tools.

### Information Sources and Search Strategy

An information specialist will search MEDLINE (Ovid), CINAHL (EBSCO), and the Cochrane Database of Systematic Reviews to identify eligible SRs published between January 1, 2023, and December 31, 2025. Two coordinated searches will be conducted: (1) a search combining validated SR filters with AI-related terminology to identify SRs explicitly reporting AI assistance; and (2) a broader search applying only the SR filter to identify the comparator sampling frame.

Validated SR filters from Canada’s Drug Agency Search Filters Database will be used and adapted as needed for each database, including the SR/meta-analysis/health technology assessment/indirect treatment comparison filter for MEDLINE and the corresponding filter for CINAHL [18]. The Cochrane Database of Systematic Reviews will be searched without an SR filter, as it exclusively indexes review articles. Search strategies will be drafted, piloted, and peer reviewed using the Peer Review of Electronic Search Strategies (PRESS) [19] guideline. All searches will be rerun prior to final analyses to ensure retrieval of newly indexed records. Database-specific search strategies for the AI-assisted SR search are provided in Multimedia Appendix 2.

### Sampling, Matching, and Study Selection

Study selection will proceed in three sequential stages: (1) identification and eligibility assessment of AI-assisted SRs, (2) construction of the traditional SR comparator sampling frame, and (3) matching and final inclusion.

#### Stage 1: Identification and Eligibility Assessment of AI-Assisted SRs

All records retrieved from the AI-targeted search will undergo a 2-stage screening process consisting of title/abstract screening followed by full-text assessment, conducted independently by 2 reviewers. Prior to each screening stage, reviewers will complete a calibration exercise on a sample of 10 records to ensure consistent interpretation of eligibility criteria; calibration will be considered acceptable at ≥80% agreement, with decision rules refined and retested if needed.

During title and abstract screening, reviewers will assess records only for article type (SR), relevance to human health, and publication characteristics (English language; 2023‐2025). No assessment or inference regarding AI use will be made at this stage. All records deemed potentially eligible based on title and abstract will undergo full-text review. During full-text assessment, reviewers will apply all inclusion and exclusion criteria and confirm explicit AI use at any stage of the SR process (eg, searching, screening, data extraction, risk-of-bias assessment, synthesis, or drafting), based on the main text, supplementary materials, acknowledgements, or disclosure statements. Discrepancies at either stage will be resolved through discussion, with unresolved cases adjudicated by a third reviewer. Ambiguous cases regarding AI involvement will prompt a single clarification email to the corresponding author. All records meeting eligibility criteria and demonstrating explicit AI involvement will constitute the AI-assisted exposure cohort.

#### Stage 2: Construction of the Comparator Candidate Pool (Unscreened)

The general SR search will provide a broad comparator sampling frame from which candidate matches will be drawn.

#### Stage 3: Matching and Final Inclusion

For each eligible AI-assisted SR, potential comparators will be identified from the traditional SR sample using the matching criteria described in “Matching Strategy.” When more than 2 potential matches are available, selection will occur using a randomized, reproducible procedure. Subsequently, 2 reviewers will screen full texts to confirm eligibility criteria are met. Comparator SRs failing eligibility will be replaced with the next randomly selected candidate. Sampling will proceed consecutively by earliest publication date within each quarter until at least 150 AI-assisted SRs and 300 matched traditional SRs have been included.

### Data Extraction

Two reviewers will independently extract all data using a piloted and standardized extraction form. Prior to formal extraction, reviewers will complete a calibration exercise on a sample of 5‐10 SRs to ensure consistent interpretation of extraction items and coding rules. Discrepancies during extraction will be resolved through discussion, with unresolved differences adjudicated by a third reviewer. Extracted data will include bibliographic characteristics; publication features; methodological elements (eg, protocol registration, number and type of included studies, and use of risk-of-bias tools); structural indicators of review oversight (eg, Cochrane affiliation, funding source, and team size); author-reported involvement of an information specialist when explicitly stated; and indicators of timeliness and dissemination. For AI-assisted SRs, additional fields will capture the AI tool name; model type (eg, large language model–based systems, screening classifiers, and others); stage of integration; prompting or configuration details when available; and any description of human oversight, validation, or verification procedures. When available, prompting or configuration details and descriptions of human oversight will be extracted to support classification of the depth of AI integration (core methodological vs supporting use) and to characterize transparency of AI reporting. Extraction items, operational definitions, and coding guidance are provided in Multimedia Appendix 3.

### Appraisal of Methodological Rigor and Reporting Completeness

Methodological and reporting quality will be appraised using AMSTAR 2 and PRISMA 2020, with ROBIS applied when relevant to assess risk of bias in the review process. No study-level risk-of-bias tools (eg, ROB 2 [Risk of Bias 2; Cochrane] and ROBINS-I [Risk of Bias in Nonrandomized Studies of Interventions; Cochrane]) will be used, as the unit of analysis is the SR. All quality assessments will be conducted in duplicate, with discrepancies resolved by consensus or third-reviewer adjudication. AMSTAR 2 and PRISMA 2020 emphasize transparency, completeness, and appropriateness of methods; therefore, high scores may still coexist with inaccuracies in extracted results. These considerations will be incorporated into the interpretation of findings.

### Statistical Analysis

#### Descriptive Analyses

Study characteristics will be summarized by exposure group (AI-assisted vs traditional SRs). Continuous variables will be reported as means with SDs or medians with IQRs, and categorical variables as frequencies and percentages. Distributions of AMSTAR 2, PRISMA 2020, ROBIS, and AITDI scores will be visualized using boxplots and density plots.

#### Primary Analysis

The primary objective is to evaluate whether the methodological quality of AI-assisted SRs is noninferior to that of traditional SRs. A linear regression model will estimate the mean difference in AMSTAR 2 total score (AI minus comparator) using a noninferiority margin of –1.0, with cluster-robust standard errors to account for matched sets. Models will adjust for prespecified confounders conceptually linked to SR quality and AI adoption, including journal impact tier, funding source (industry vs nonindustry), Cochrane affiliation, team size, and review question type (eg, intervention, diagnostic, and prognostic). The modified AMSTAR 2 total score will be treated as an approximately continuous outcome. Score distributions will be examined using histograms and density plots to assess skewness and floor or ceiling effects. Linear regression was selected because the primary inferential goal is estimation of an absolute difference in average methodological adherence between groups on a directly interpretable scale.

The noninferiority margin of −1.0 was selected a priori to represent the largest acceptable decrement corresponding approximately to one AMSTAR 2 item-level adherence difference on the 13-point modified scale. Differences greater than this threshold would be considered potentially meaningful from a methodological perspective. Because additive scoring does not fully reflect the intended critical-domain structure of AMSTAR 2, results from the primary analysis will be interpreted alongside complementary analyses of AMSTAR 2 overall confidence ratings and prespecified critical domains.

We acknowledge that key drivers of SR quality, such as author team expertise, information specialist involvement, and journal-level enforcement of PRISMA standards, may not be consistently or objectively ascertainable from published reports, and that residual confounding may persist. To mitigate this, our models adjust for established and directly extractable proxies of review rigor, including journal impact tier, Cochrane affiliation, funding source, team size, and review question type. Information specialist involvement will be summarized descriptively and, where reporting is sufficiently complete, it will be incorporated into exploratory adjusted models. Matching variables (review type, meta-analysis status, clinical domain, and publication year) will be added only if residual imbalance persists (standardized mean difference >0.10).

#### Secondary Analyses

The following secondary analyses are planned:

(1) AMSTAR-2 domain-based analyses: to complement the modified item-level adherence analysis and better reflect the intended use of AMSTAR 2, overall confidence ratings (high, moderate, low, or critically low) will be summarized by group and compared using ordinal logistic regression with cluster-robust variance estimators or multinomial models if the proportional odds assumption is violated. Prespecified critical AMSTAR 2 domains will also be summarized descriptively and compared between groups using binary regression models where appropriate.

(2) Reporting quality and risk-of-bias rigor: this will be evaluated using linear regression for PRISMA 2020 adherence and ordinal or multinomial logistic regression for ROBIS judgments, with all models applying cluster-robust variance estimators and adjusting for the same core covariates as in the primary analysis.

(3) AI transparency (AITDI): for AI-assisted SRs, AITDI scores will be summarized descriptively (eg, means, medians, and distribution plots) to characterize the transparency and disclosure practices. Exploratory, hypothesis-generating analyses will examine whether AITDI scores vary by journal impact tier, funding source, review-group affiliation (eg, Cochrane), review type, and depth of AI integration, using univariable and multivariable regression models as appropriate and restricted to AI-assisted reviews. In Aim 1, AITDI will be treated as a preliminary instrument and used primarily as an explanatory variable in exploratory models of SR quality. Formal refinement, reliability testing, and validation of AITDI will be conducted in Aim 2 and reported separately.

(4) Timeliness and dissemination will be assessed using linear regression or accelerated failure-time models for timeliness (days from protocol registration or earliest submission to publication) and negative binomial or linear regression for bibliometric impact (12-month citation counts and Altmetric Attention Scores). Because these outcomes are influenced by different mechanisms than methodological quality, models will adjust for outcome-specific covariates such as journal tier, publication year, and topic area rather than the core methodological confounders used in earlier analyses.

(5) Predictors of SR quality: exploratory multivariable models will assess characteristics associated with higher AMSTAR 2 and PRISMA 2020 scores, including review type, review question type, team size, journal tier, and clinical domain. These analyses will be hypothesis-generating. Among AI-assisted SRs, exploratory models will also examine whether higher AITDI transparency scores are associated with higher AMSTAR 2 and PRISMA 2020 scores, recognizing that AITDI is a provisional instrument at this stage.

(6) Depth of AI integration: among AI-assisted SRs, reviews using AI for core methodological tasks (screening, extraction, or risk-of-bias assessment) will be compared with those using AI only for supporting or reporting functions. Mean differences in AMSTAR 2 and PRISMA 2020 scores will be estimated using adjusted regression models with cluster-robust variance.

#### Sensitivity Analyses

Several robustness checks will be conducted: (1) matched-set fixed-effects models to control for within-pair confounding; (2) propensity-weighted analyses using overlap weights on journal tier, funding source, and team size; (3) reclassification analyses restricting to reviews with clearly documented AI use in core methodological stages; (4) leave-one-journal-out analyses to assess the influence of high-volume outlets; (5) analyses restricted to standard (nonliving) systematic reviews to assess whether inclusion of living reviews influences the estimated association between AI assistance and methodological quality; (6) alternative specifications of methodological quality, including proportional odds ordinal logistic regression using AMSTAR 2 overall confidence categories and analyses restricted to prespecified critical domains only; and (7) to examine the potential impact of exposure misclassification due to undisclosed AI use, we will perform a sensitivity analysis restricted to comparator SRs with an explicit statement of no AI use.

#### Missing Data

Missing methodological or bibliometric variables will first be recovered from supplementary files or registered protocols; if unresolved, authors will be contacted once. Remaining missing data will be handled via multiple imputation by chained equations, with complete-case analysis as a sensitivity check.

### Sample Size and Power

This study is powered to assess noninferiority in methodological quality between AI-assisted and traditional SRs using the modified AMSTAR 2 adherence scores. Prior methodological evaluations comparing AI-assisted and non-AI reviews report AMSTAR 2 totals typically in the 6‐8 range with no significant differences between groups, supporting our assumption of a true mean difference close to zero and a SD of approximately 2.5‐3.0 [7]. We define a noninferiority margin of −1 point (AI minus comparator). Using a one-sided *α*=.025 and a planned sample of 150 AI-assisted reviews and 300 matched comparators (1:2 allocation), the study has approximately 98% power to demonstrate noninferiority under these assumptions. All power calculations were performed in R (R Foundation for Statistical Computing).

The number of eligible AI-assisted SRs is not known a priori. We will include all eligible AI-assisted reviews identified within the prespecified sampling frame, up to a target of 150. If fewer than 150 AI-assisted reviews are identified, all eligible reviews will be included, and the achieved precision and power will be reported. If yield is insufficient, a prespecified, stepwise expansion of the search (eg, extending the publication window and/or information sources using identical eligibility criteria and exposure definitions) will be undertaken and fully documented. If more than 150 eligible AI-assisted reviews are identified, inclusion will be capped at 150 unless additional inclusion is required to maintain matching balance or improve precision, in which case this will be specified a priori. The relative use of LLMs versus other AI tools (eg, screening classifiers) will be determined empirically during data extraction.

### Aim 2: Development, Refinement, and Evaluation of the AITDI

#### Rationale and Work to Date

AI use in SRs is often poorly disclosed, with important details (tool identity, model version, stage of use, prompts, or oversight) frequently omitted, limiting reproducibility and bias appraisal [20]. We developed a preliminary version of the AITDI (Table 1) as a minimal transparency index designed to support meta-research (not a reporting guideline). Aim 2 will refine and validate the AITDI using Moher et al’s [15] reporting guideline development guidance.

#### Phase 1: Preparatory Evidence Mapping and Initial Item Generation

**Figure 4.**
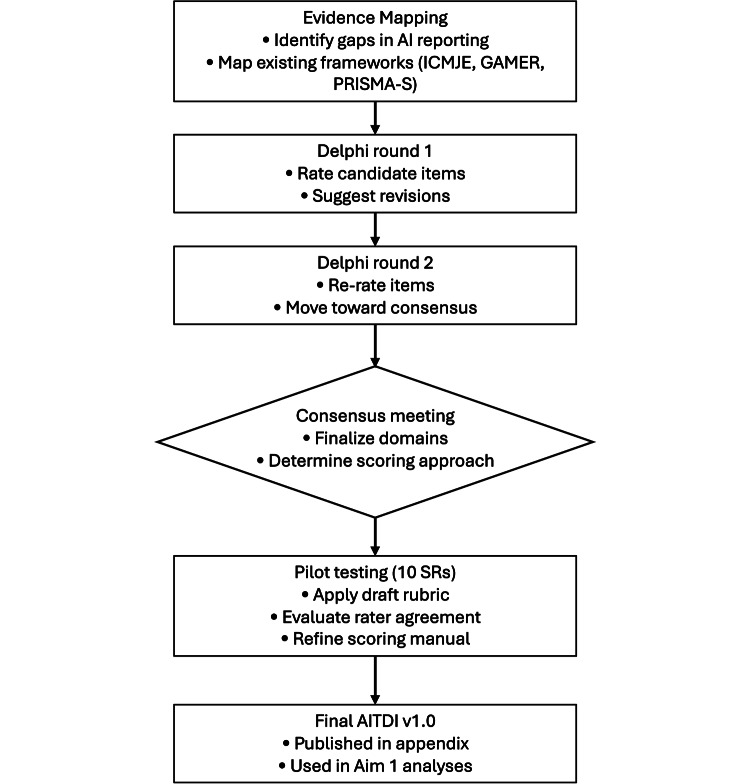
Development process for the AI Transparency and Disclosure Index (AITDI). Steps include scoping, Delphi rounds, a consensus meeting, pilot testing, and finalization. AI: artificial intelligence; AITDI: AI Transparency and Disclosure Index; GAMER: Guidelines for AI in Medical Evidence Reporting; ICMJE: International Committee of Medical Journal Editors; PRISMA-S: Preferred Reporting Items for Systematic Reviews and Meta-Analyses–Search; SR: systematic review.

We have started preparatory, concept-driven evidence mapping to identify common transparency gaps in published AI-assisted systematic reviews and to situate these gaps within the context of existing reporting expectations. This work involved targeted review of EQUATOR (Enhancing the Quality and Transparency Of health Research)-listed reporting guidelines [21] and extensions relevant to AI and SR (PRISMA-S [PRISMA–Search extension] [22], CONSORT-AI [Consolidated Standards of Reporting Trials–Artificial Intelligence extension], and SPIRIT-AI [Standard Protocol Items: Recommendations for Interventional Trials–Artificial Intelligence extension] [23]), alongside review of policy-level guidance (eg, International Committee of Medical Journal Editors AI updates [24]) and recent scholarly proposals addressing AI transparency in medical research and systematic reviews (Generative Artificial Intelligence tools in Medical Research [25], PRISMA-trAIce [PRISMA–Transparent Reporting of Artificial Intelligence in Comprehensive Evidence Synthesis] [26]). This phase will conclude with the consolidation of all candidate items into an initial checklist that will undergo structured evaluation in Phase 2. Figure 4 outlines the multistep development process for the AITDI, including preparatory evidence mapping, Delphi surveys, consensus activities, pilot testing, and finalization.

#### Phase 2: Premeeting Delphi Exercise and Item Reduction

A 2‐3 round online Delphi survey [27] will be conducted to refine and prioritize candidate AITDI items. Participants will be purposively recruited to form a multidisciplinary expert panel, including SR authors and methodologists (ie, researchers who conduct and publish SRs and are intended end users of the tool), AI/ML experts, information specialists, guideline developers, journal editors, clinicians, and patient partners. Eligible panelists will have demonstrated experience in conducting or publishing systematic reviews, AI-assisted research, reporting guideline development, editorial or peer-review activities, or use of systematic reviews in practice. We anticipate recruiting approximately 20‐40 panelists (consistent with best practices for Delphi studies and reporting guideline development). Panelists will rate each candidate item for importance and clarity using a structured Likert scale and may suggest item refinement, consolidation, or removal. After each round, anonymized summary statistics (including median ratings and rating distributions) and synthesized qualitative feedback will be shared with participants. A priori-defined consensus criteria will be used to guide item retention, modification, or removal across rounds, and results from the Delphi process will be used to prioritize items for discussion at the subsequent consensus meeting.

#### Phase 3: Consensus Meeting

The consensus meeting will convene a purposively selected subset of participants from the Delphi exercise to ensure balanced representation across the relevant knowledge user groups and end user perspectives. Approximately 15‐25 participants will be invited, selected based on Delphi participation, demonstrated expertise, and representation across disciplines, with balanced representation across knowledge users. The meeting will focus on finalizing the AITDI checklist as a transparency assessment instrument, including discussion of the rationale, conceptual necessity, and transparency value of each candidate item, as well as development of a conceptual schema to visualize AI use across the SR workflow. Decisions will be informed by the Delphi results and relevant evidence summaries, with structured discussion and voting used to resolve disagreements regarding the content, structure, and presentation of the AITDI items. The meeting will also define next steps related to finalizing the index, including drafting responsibilities, authorship, and a knowledge translation strategy for dissemination of the AITDI.

#### Phase 4: Drafting, Piloting, and Reliability Assessment

The final draft AITDI statement and an accompanying explanation and elaboration (E&E) document will be prepared. The AITDI statement will outline the scope, development process, and final checklist. The E&E document will provide a detailed rationale for each checklist item, with illustrative examples from the literature and empirical justification for inclusion. The checklist and E&E document will be pilot-tested using a purposive sample of 20 AI-assisted systematic reviews identified in Aim 1. Two independent raters will apply the AITDI to these reviews to assess usability, clarity, and interrater agreement. In addition, raters will provide brief structured qualitative feedback (eg, short open-ended comments) on item interpretability, feasibility of application, and perceived burden. Quantitative agreement metrics and qualitative feedback will be synthesized to inform final refinements to the AITDI [28]. Insights from Aim 1 regarding current reporting gaps and patterns of AI use, along with quantitative reliability metrics and qualitative feedback from Phase 4 pilot testing, will inform final refinements to the AITDI.

#### Phase 5: Dissemination and Updating Strategy

We will submit the AITDI statement and E&E document for peer-reviewed publication. We will also make the AITDI checklist, scoring manual, and example applications publicly available on an open-access platform, such as Open Science Framework (OSF), and seek inclusion of the AITDI in the EQUATOR Network’s database of reporting guidelines for AI in SRs. A plan for updating the AITDI will be developed, with regular reviews scheduled to ensure that the index remains relevant as AI tools and disclosure standards evolve.

### Aim 3: Reproducibility of AI-Generated Outputs

#### Study Overview

Aim 3 evaluates the reproducibility of AI performance across (1) repeated runs of the same model, (2) different AI models, and (3) AI compared with human reviewers. Unlike Aim 1, which evaluates completed SRs, Aim 3 focuses on task-level reproducibility, treating each SR task as a rating exercise analogous to human interrater reliability studies. Task sets will be derived from SRs included in Aim 1 to ensure diversity in review types, clinical domains, and methodological quality, and to enable construction of high-quality human consensus reference standards. Aim 3 is intentionally restricted to task types for which reproducibility can be meaningfully operationalized. Specifically, included tasks must have (1) discrete, well-defined inputs, (2) a constructible human consensus reference standard, and (3) evaluable agreement using established reliability metrics analogous to inter-rater reliability. Screening decisions, risk-of-bias assessments, data extraction, and evidence summarization meet these criteria and, therefore, allow direct comparison across repeated runs, AI models, and human reviewers. Other AI-assisted activities (eg, search strategy generation or narrative drafting) are conceptually important but do not satisfy these conditions in a way that supports formal reproducibility testing; accordingly, they are not examined through reproducibility metrics in Aim 3.

#### Selection of SRs for Task Set Construction

**Figure 5.**
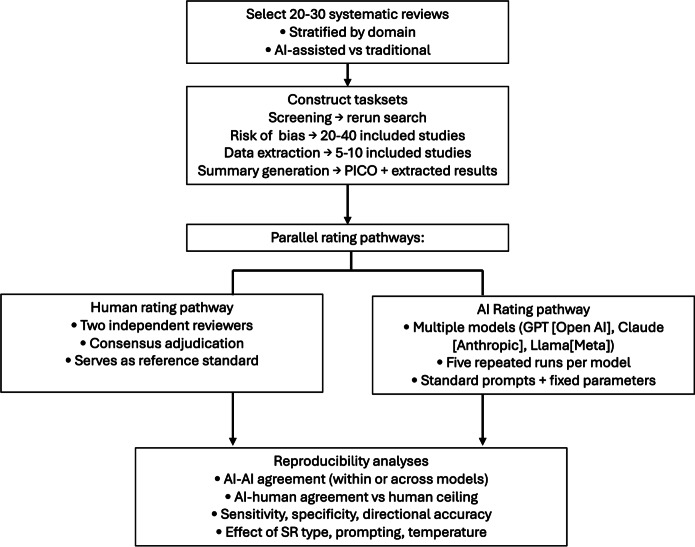
Workflow for reproducibility task set construction and parallel human and AI rating pathways. AI: artificial intelligence; PICO: population, intervention, comparator, and outcome; SR: systematic review.

A random, stratified subset of 20‐30 SRs from Aim 1 (stratified by clinical domain and AI-assisted vs traditional status) will be used to generate task sets. Figure 5 illustrates the workflow for constructing reproducibility task sets and conducting parallel human and AI rating pathways. For all task sets, human raters will undergo standardized training on task-specific instructions and instruments prior to formal data collection. A calibration exercise will be conducted for each task type using a small subset of records or studies. During formal task execution, disagreements between raters will be resolved by consensus or adjudication by a third reviewer, as appropriate, to generate the human reference standard used for comparison with AI outputs.

### Task Types

The following task types will be evaluated to assess reproducibility across AI-assisted systematic review workflows:

1. Screening decisions: Each task set will include 200‐400 titles/abstracts per SR, constructed by combining all included studies from the SR with additional records retrieved by rerunning the authors’ search. Two independent reviewers will apply the SR’s eligibility criteria to all records, with disagreements resolved by consensus to form the human reference standard. AI models (eg, GPT-based [OpenAI], Claude [Anthropic PBC], and Llama [Meta Platforms]) will screen the same task sets using standardized prompt templates and standardized generation parameters (see “AI Model Selection, Prompt Standardization, and Configuration”). Each model will be run five times per task set to capture nondeterministic variability in generative outputs while maintaining feasibility across tasks and models; prior evaluations of LLM-assisted citation screening demonstrate measurable run-to-run variability, supporting the use of repeated executions when assessing reproducibility and agreement [29,30].
2. Risk-of-bias assessments: for each SR, 20-40 included studies will be sampled. Two blinded human raters will conduct domain-level risk-of-bias assessments using the same instrument specified in the original SR (eg, RoB 2 for randomized trials and ROBINS-I for nonrandomized studies, as applicable). AI models will generate domain-level risk-of-bias ratings with required justifications across repeated runs using standardized prompts aligned to the same instruments and response options used by human raters (see “AI Model Selection, Prompt Standardization, and Configuration”).
3. Data extraction: for each SR, a purposive sample of 5-10 included studies will be selected to reflect variation in study design, outcome type (eg, binary, continuous, or time-to-event), and reporting complexity (eg, clearly reported vs ambiguous data). This range is sufficient to evaluate the reproducibility of data extraction across key quantitative variables while maintaining feasibility, given that the objective is reproducibility assessment rather than exhaustive extraction. Human extraction will occur in duplicate, while AI extraction will be repeated across models and runs using the same standardized extraction prompts and output schema.
4. Summary and conclusion generation: task sets will include PICO elements and the human reference quantitative results from Task 3. Two human reviewers will independently generate narrative summaries. AI models will generate summaries using standardized prompt templates and standardized generation parameters, and responses will be coded for correctness in direction of effect, magnitude or interpretation, and certainty language.

### AI Model Selection, Prompt Standardization, and Configuration

Selection of AI models for Aim 3 will follow transparent criteria rather than a fixed list of systems to ensure that the study reflects the range of contemporary LLMs available at the time of evaluation. To support reproducibility across repeated runs and across models, standardized prompt templates and output schemas will be developed for each task type before formal evaluation. Final prompt templates, task instructions, and output schemas will be archived on the project’s OSF repository to support reproducibility. Models will be chosen to represent distinct classes of contemporary LLMs relevant to SR workflows (eg, closed-source commercial, independently developed commercial, and open- or partially open-source systems). Eligible models must be externally available (ie, not bespoke or internally developed), capable of processing the required task inputs, and permit documentation of generation settings sufficient to support reproducibility assessment.

For each task type, we will develop a prompt template before formal testing, which will be standardized in structure, instructions, and requested output format, with only task-specific fields varying across task sets (eg, eligibility criteria, risk-of-bias instrument, extraction fields, or PICO elements). Prompts will mirror the information available to human reviewers and will request only final structured decisions (eg, classification or extraction outputs) without generating chain-of-thought reasoning. Where model-specific formatting adjustments are required (eg, differences in system prompt syntax or input formatting), these adaptations will only be limited to formatting changes and will not modify the substantive task instructions or requested outputs.

All models will receive the same source materials within each task set, presented in the same order and format as far as platform constraints allow. For both repeated-run and cross-model analyses, the same prompt template, task instructions, target output schema, and generation settings will be applied without modification wherever technically feasible. Prompt templates will be pilot tested on a small subset of task sets before formal evaluation to optimize clarity and confirm feasibility; no further substantive prompt changes will be made once formal data collection begins. Any unavoidable deviations from the prespecified prompt templates will be reported.

Model names, versions, access conditions, dates of use, and generation settings (eg, temperature or equivalent stochasticity controls, output-length limits, and other available stability parameters) will be documented before analysis and applied consistently across task sets. No additional pretraining, fine-tuning, or model adaptation will be performed as part of this study; all LLMs will be used in their externally available configurations. Generation parameters (eg, temperature or equivalent controls) will be fixed across repeated runs and across models where technically feasible to support reproducibility.

### Reproducibility Metrics

Reproducibility will be evaluated using the following complementary metrics and comparative analyses:

- Reproducibility Metrics
- AI-AI reproducibility: agreement will be assessed across repeated runs of the same model and across different models using Cohen κ, Fleiss κ, or intraclass correlation coefficient (ICC), as appropriate.
- AI-human agreement: human consensus will serve as the reference standard for all tasks. Agreement, sensitivity, specificity, and directional concordance will be calculated using the same reliability metrics applied to human-human comparisons (eg, Cohen κ, weighted κ, ICC) and interpreted relative to task-specific human reliability ceilings.
- Human-human reliability: two independent raters per task will be assessed using the same interrater reliability metrics (eg, Cohen κ, weighted κ, or ICC, as appropriate) to establish the reliability ceilings for each task.
- Exploratory comparisons: additional analyses will assess whether reproducibility differs for task sets derived from AI-assisted versus traditional systematic reviews and whether variations in prompting structure or model parameters influence reproducibility. These analyses will include comparisons of repeated runs conducted at low temperature versus default generation settings, where supported by the model.

For selected tasks that generate free-text outputs (eg, narrative summaries, risk-of-bias justifications, or conclusion statements), we will conduct a secondary, exploratory semantic concordance analysis using an independent LLM as an automated evaluator (“LLM-as-a-judge”) [31,32]. This component is intended only to assess whether paired outputs are substantively similar in meaning when conventional structured agreement metrics are insufficient, and it will not replace the primary reproducibility analyses based on κ- or ICC-based reproducibility estimates.

The judge model will evaluate paired outputs using a prespecified categorical rubric (eg, semantically equivalent, partially equivalent, or discordant). Judge prompts will be standardized, and the judge model will be instructed to assess semantic concordance rather than stylistic similarity. Where feasible, outputs will be deidentified and presented in random order to reduce bias related to source attribution (eg, model identity or human vs AI origin). We will document the judge model, model version, access date, and evaluation prompt and interpret findings in light of known limitations of LLM-as-a-judge approaches [31,32].

As the LLM-as-a-judge approach may introduce additional model-dependent biases, this analysis will be treated as exploratory and will be benchmarked against independent human assessment of a random 10%‐20% sample of paired outputs, consistent with prior automation-assisted validation approaches in SR research [33]. Two human reviewers will apply the same semantic concordance rubric, with disagreements resolved by discussion or third-reviewer adjudication. Human rating will be used to contextualize agreement estimates from the judge model, and any material discrepancies between human and LLM-derived semantic judgments will be reported. Results from the LLM-as-a-judge analysis will be presented descriptively and interpreted cautiously.

### Aim 4: Qualitative Component

#### Qualitative Study Design

We will conduct a qualitative study using interpretive description, situated within a constructivist paradigm, to explore how diverse knowledge users conceptualize rigor, transparency, and accountability in AI-assisted SRs. A constructivist approach is appropriate given the absence of a single objective standard for acceptable AI use in SR and the expectation that norms of rigor and trustworthiness are socially constructed and context-dependent [24,34]. This aim complements the quantitative and experimental components (Aims 1‐3) by examining why gaps in reporting and reproducibility arise and what conditions knowledge users view as acceptable, unsafe, or in need of governance.

#### Participants and Sampling

We will use purposive, maximum-variation sampling to recruit approximately 25‐30 participants across six knowledge user groups, including (1) SR authors and methodologists (including faculty researchers, graduate students, and research-active medical trainees who conduct, contribute to, peer-review, or critically use SRs, even if they do not primarily publish SRs), (2) journal editors and peer reviewers, (3) guideline developers and health technology assessment committee members, (4) clinicians (including practicing physicians and clinically focused trainees, such as residents and fellows) who use SRs in practice, (5) policymakers, funders, or organizational knowledge users, and (6) patient partners involved in research, guideline processes, or advisory roles. Eligibility criteria include age ≥18 years, English proficiency, self-reported familiarity with SRs (eg, through authorship, peer review, guideline development, clinical use, or research training), and willingness to participate in a 45‐60-minute interview. Individuals recruited through a direct clinician-patient relationship with study investigators will be excluded. Sampling will continue until thematic saturation is reached within each knowledge user group, defined as the point at which no new themes emerge [35,36]. We will target 5‐8 participants per knowledge user group (minimum 5 where feasible), with iterative monitoring of thematic sufficiency within and across groups [37]. Sample sizes may be adjusted pragmatically to ensure diversity of perspectives.

#### Recruitment and Consent

Participants will be identified through professional networks (eg, Cochrane and SR methods groups), author lists from Aims 1 and 3, editorial boards, guideline and health technology assessment committees, institutional knowledge user networks, and patient engagement organizations. Snowball sampling will be used to identify additional eligible participants. Interested individuals will receive an information sheet and will provide verbal or electronic informed consent prior to the interview. Consent will emphasize participants’ rights to withdraw at any time and that their responses will remain confidential. Participants will be offered a modest honorarium in recognition of their time and in accordance with institutional policies.

### Data Collection

#### Overview

Data will be collected through semi-structured interviews (approximately 45‐60 minutes) conducted via secure videoconferencing (eg, Zoom [Zoom Video Communications Inc] for health care) or telephone. The interview guide will be informed by findings from Aims 1‐3 and will explore the following: (1) participants’ experience with SR and/or AI tools; (2) perceived benefits and risks of AI-assisted SRs across key workflow stages (searching, screening, extraction, appraisal, and summarization); (3) expectations for AI disclosure, human oversight, and accountability; and (4) conditions under which AI-assisted SRs would be considered sufficiently rigorous and trustworthy for clinical, policy, or patient-facing use. Interviews will be audio-recorded, professionally transcribed, and deidentified. The interview guide will be pilot-tested with 1‐2 participants and iteratively refined based on feedback, and data collection will continue until thematic sufficiency is achieved across all knowledge-user groups. The full interview guide is provided in Multimedia Appendix 4.

#### Data Analysis and Rigor

We will use reflexive thematic analysis (Braun and Clarke [38]) to analyze interview transcripts, supported by qualitative data analysis software (eg, NVivo [Lumivero]). The analytic process will include: (1) familiarization with the data, (2) initial coding, (3) development and refinement of a coding framework, and (4) iterative theme generation and review. Two investigators will independently code early transcripts to calibrate coding practices before proceeding to more flexible single-coding with regular analytic meetings. Rigor strategies will include reflexive journaling, peer debriefing within the investigator team, and light-touch member checking (eg, sharing a brief thematic summary with a small subset of participants to confirm resonance). Reporting will follow the Consolidated Criteria for Reporting Qualitative Research (COREQ) guidance [39] to ensure rigor in qualitative research reporting.

#### Integration With Other Aims

Qualitative findings will be used to (1) interpret quantitative differences in methodological quality and transparency identified in Aim 1, (2) contextualize reproducibility patterns observed in Aim 3, and (3) refine and prioritize AITDI domains and items in Aim 2, particularly around expectations for AI disclosure, human oversight, and acceptable versus unacceptable AI uses in SR workflows.

### Ethical Considerations

Aims 1 and 2 (comparative quality assessment of published systematic reviews and development of the AI transparency and disclosure index) and Aim 3 (reproducibility testing using task sets derived from published reviews) involve secondary analysis of publicly available, deidentified data and simulated rating exercises using published materials. In accordance with institutional policy, these components qualify for exemption from research ethics board review.

Aim 4 (qualitative interviews with knowledge users) has been submitted to the Sunnybrook Research Ethics Board (approval pending at the time of manuscript revision; study ID: 7101). All interview participants will provide informed consent prior to participation. For the qualitative component, participants will receive an information sheet outlining study procedures, risks, and benefits, and consent will be obtained electronically or verbally prior to data collection. Participation is voluntary and participants may withdraw at any time without consequence. Audio-recorded interviews will be professionally transcribed and deidentified prior to analysis. Transcripts and analytic files will be stored on secure, password-protected institutional servers accessible only to authorized study personnel. Only anonymized or aggregated qualitative findings will be publicly reported. Participants in Aim 4 will receive an honorarium in recognition of their time and contributions.

### Patient Involvement and Dissemination

Patient and clinician partners will contribute to protocol refinement, interpretation of qualitative and quantitative findings, and development of accessible dissemination materials. Their involvement will be reported using Guidance for Reporting Involvement of Patients and the Public version 2 (GRIPP2) [40]. Honoraria will be offered when feasible and appropriate. Study findings will be disseminated through peer-reviewed publications, conference presentations, and public repositories. An open-access AI transparency checklist for SR authors, editors, and guideline developers will be developed based on study results and made available through the project’s OSF page. This strategy aims to support transparent and responsible use of AI in SR.

## Results

As of December 2025, this study has been preregistered on the OSF (DOI: 10.17605/OSF.IO/Q5JRW), the search strategy has been finalized, and title/abstract screening has begun. Full-text screening and data extraction are planned for Feb-April 2026, followed by refinement and application of the AITDI and reproducibility testing from May to October 2026. Qualitative interviews are anticipated from October 2026 to February 2027. Final analyses and synthesis of quantitative and qualitative findings are expected to be completed by April 2027, with dissemination planned for mid-2027.

## Discussion

The integration of AI into SR workflows has accelerated rapidly. However, methodological and reporting frameworks used to appraise review quality have not kept pace [2]. Existing evaluations have largely focused on isolated tasks (eg, screening) or narrow clinical domains, with limited attention to the overall methodological rigor, reporting completeness, transparency, and reproducibility of AI-assisted SRs [7-9]. In addition, AI use is often poorly documented, making it difficult to assess where and how tools were applied, what safeguards were in place, and whether outputs are reproducible [33]. This protocol evaluates the methodological quality, transparency, and reproducibility of AI-assisted SRs and explores knowledge-user expectations for acceptable use and disclosure.

This study has several methodological strengths. First, the matched comparative design in Aim 1 controls for publication year, topic area, review type, and meta-analysis status, reducing confounding from field differences and time-based trends. Second, dual independent scoring with validated appraisal (AMSTAR 2, ROBIS) and reporting tools (PRISMA 2020), combined with a structured AI transparency rubric (AITDI), provides a rigorous and reliable framework for evaluating both methodological quality and the completeness of AI-related reporting. Third, the reproducibility component (Aim 3) moves beyond single-run performance evaluations by examining agreement across repeated runs of the same model, across different models, and between AI and human reviewers, while benchmarking AI-human agreement against human-human reliability. Finally, the qualitative component (Aim 4), grounded in interpretive description within a constructivist paradigm, ensures that the quantitative and experimental findings are interpreted in light of how authors, editors, guideline developers, clinicians, policymakers, and patient partners conceptualize acceptable versus unsafe AI use, disclosure, and accountability in SRs.

There are also important limitations. Identification of AI-assisted reviews will depend on explicit reporting in manuscripts, supplementary materials, acknowledgements, or AI disclosure statements. As a result, underreporting may lead to misclassification and likely underestimates the true prevalence and depth of AI use in review workflows. Some reviews classified as traditional comparators may therefore have involved unreported AI use. Because currently available AI-detection tools primarily assess stylistic features rather than workflow-level methodological use, they are not sufficiently reliable for identifying undisclosed AI assistance in published reviews. If such misclassification is nondifferential with respect to methodological quality, it would be expected to bias effect estimates toward the null and reduce our ability to detect true between-group differences. However, disclosure of AI use may also correlate with other characteristics, such as author transparency, journal reporting standards, or methodological rigor. Accordingly, the comparison in Aim 1 should be interpreted as primarily reflecting reviews with reported AI use versus reviews without reported AI use, rather than a definitive causal contrast between truly AI-assisted and truly non–AI-assisted reviews. Furthermore, quality assessment focuses on methodological and reporting standards rather than verifying numerical accuracy of extracted data or meta-analytic calculations, and unmeasured differences between AI-assisted and traditional reviews may remain despite matching and adjustment. Restriction to English-language health and biomedical journals may limit generalizability. In Aim 3, reproducibility estimates are based on a subset of contemporary models and tasks; as AI tools evolve, absolute performance may change. Residual confounding from unmeasured factors (eg, author team expertise or internal review processes) may persist. Estimates of the association between AI assistance and methodological quality will be interpreted cautiously. Incomplete reporting of prompting strategies or model configuration may further limit characterization of AI use. Moreover, in Aim 3, observed reproducibility may be influenced not only by underlying model behavior but also by prompt design and platform-specific implementation constraints; although prompts and generation settings will be standardized and documented, some residual prompt-related variability may remain. Finally, given that AI tools and reporting norms are evolving rapidly, findings from this 2023‐2025 sampling frame should be interpreted as reflecting an early phase of LLM integration into SR workflows.

Analogous to psychometric properties used to evaluate clinical tests (eg, sensitivity, specificity, and reliability), reproducibility estimates (eg, within-model stability across repeated runs, between-model agreement, and benchmarking against human-human variability) could represent a candidate set of performance characteristics that AI tools should report when used in SRs. While defining acceptable thresholds is beyond the scope of the present study, the empirical framework applied in Aim 3 illustrates how such metrics can be generated and interpreted and may inform future standards, journal policies, or guidance on acceptable AI use in SRs.

Despite these constraints, this study is well positioned to make several contributions. By providing empirical estimates of methodological and reporting quality for AI-assisted versus traditional SRs, it will clarify whether this AI integration is neutral or detrimental to review rigor. The AITDI is expected to offer a practical, structured instrument for documenting and appraising AI use, which can inform journal policies, editorial expectations, and future AI-specific extensions of existing tools (eg, PRISMA-LLM [PRISMA–large language model] and A Measurement Tool to Assess Systematic Reviews-large language model [AMSTAR-LLM]). Reproducibility analyses will help delineate when AI tools can be treated as reasonably stable co-reviewers and when tighter human oversight or additional safeguards are warranted. The qualitative findings will complement these results by illuminating how different knowledge users define rigor, transparency, and trustworthiness in AI-assisted SRs and by identifying perceived “red lines,” acceptable use cases, and governance priorities.

This work is intended as a foundational step toward transparent, accountable, and methodologically robust use of AI in SR. As AI capabilities expand and disclosure expectations evolve, the framework, transparency index, reproducibility assessments, and knowledge user-informed insights developed here can be updated and reapplied to ensure that innovations in SR workflows are matched by appropriate standards for quality, transparency, and oversight. Future applications could include repeat evaluations in later periods (eg, 2027 and beyond). Extensions may also examine whether AI-assisted reviews differ from traditional reviews in the framing of their conclusions, building on prior meta-epidemiologic work evaluating conclusion optimism and interpretive “spin” in SRs [41].

## Supplementary material

10.2196/90588 Multimedia Appendix 1 Summary of primary and secondary outcomes and quality assessment tools.

10.2196/90588 Multimedia Appendix 2 Full search strategies for MEDLINE, Cochrane, and CINAHL (Executed December 3-5, 2025).

10.2196/90588 Multimedia Appendix 3 Data extraction variables and coding guidance.

10.2196/90588 Multimedia Appendix 4 Semistructured interview guide.

## Abbreviations

AI: artificial intelligence
AITDI: AI Transparency and Disclosure Index
AMSTAR 2: A Measurement Tool to Assess Systematic Reviews version 2
AMSTAR-LLM: A Measurement Tool to Assess Systematic Reviews-Large Language Model
CONSORT-AI: Consolidated Standards of Reporting Trials-artificial intelligence extension
COREQ: Consolidated Criteria for Reporting Qualitative Research
E&E: explanation and elaboration
EQUATOR: Enhancing the Quality and Transparency of Health Research
GRIPP2: Guidance for Reporting Involvement of Patients and the Public version 2
ICC: intraclass correlation coefficient
LLM: large language model
MeSH: Medical Subject Headings
OSF: Open Science Framework
PICO: population, intervention, comparator, and outcome
PRESS: Peer Review of Electronic Search Strategies
PRISMA: Preferred Reporting Items for Systematic Reviews and Meta-Analyses
PRISMA-LLM: Preferred Reporting Items for Systematic Reviews and Meta-Analyses for Large Language Model
PRISMA-S: Preferred Reporting Items for Systematic Reviews and Meta-Analyses-Search extension
PRISMA-trAIce: Preferred Reporting Items for Systematic Reviews and Meta-Analyses-Transparent Reporting of Artificial Intelligence in Comprehensive Evidence Synthesis
ROB2: Risk of Bias 2
ROBINS-I: Risk of Bias in Nonrandomized Studies of Interventions
ROBIS: Risk of Bias in Systematic Reviews
SPIRIT-AI: Standard Protocol Items: Recommendations for Interventional Trials-artificial intelligence extension
SR: systematic review.
