# Supplementary material for: Evaluating the Methodological Quality of Artificial Intelligence–Assisted Systematic Reviews: Protocol for a Mixed Methods Meta-Research Study
Source: JMIR Res Protoc. 2026 May 14;15:e90588. doi: 10.2196/90588 (PMC13175306; doi:10.2196/90588)
Supplement: Multimedia Appendix 1 [file resprot-v15-e90588-s001.docx]

**Multimedia Appendix 1.** Summary of primary and secondary outcomes and quality assessment tools.

**Table 1.** Primary and Secondary Outcomes

| **Outcome Type** | **Outcome** | **Description / Measurement Approach** |
| --- | --- | --- |
| **Primary Outcome** | Methodological quality (AMSTAR-2 total score) | Measured using a modified 0–13 quantitative scoring approach based on AMSTAR-2. Item-level judgments are recorded; critical domains (protocol registration, duplicate processes, search comprehensiveness, risk-of-bias assessment) summarized descriptively. Full AMSTAR-2 definitions available in Shea et al. (2017). |
| **Secondary Outcomes** | PRISMA-2020 reporting adherence | Percentage adherence across 27 reporting items. Item-level coding retained for transparency and reproducibility analyses. |
|  | ROBIS risk-of-bias rigor | Domain-level and overall ROBIS judgments for reviews conducting risk-of-bias assessment. |
|  | AI transparency (AITDI score) | Total preliminary AITDI score (0–6), capturing tool identity, stage of use, model/version reporting, prompting/configuration detail, human verification, and data governance/ethics statements. Scoring will be refined in Aim 2. |
|  | Timeliness of publication | Days from protocol registration (or earliest available milestone, e.g., submission date) to publication. |
|  | Early dissemination metrics | 12-month citation count (OpenAlex/Dimensions) and Altmetric Attention Score. |
|  | Predictors of review quality | Exploratory analyses of review type, inclusion of meta-analysis, journal tier, team size, clinical domain, and depth of AI integration. |
|  | Qualitative outcomes | Themes describing perspectives on rigor, transparency, trust, and acceptable AI use among researchers, editors, clinicians, policymakers, and patient partners (Aim 4). |

**Abbreviations:** AI: artificial intelligence; AITDI: AI Transparency and Disclosure Index; AMSTAR-2: A Measurement Tool to Assess Systematic Reviews version 2; PRISMA-2020: Preferred Reporting Items for Systematic Reviews and Meta-Analyses 2020; ROBIS: Risk of Bias in Systematic Reviews.

**Table 2.** Summary of Quality Assessment Tools

| **Tool** | **Purpose** | **Domains Assessed** | **Use in This Study** |
| --- | --- | --- | --- |
| **AMSTAR-2** | Evaluates methodological rigor of systematic reviews. | 16 items covering protocol registration, search quality, duplicate processes, risk-of-bias assessment, and synthesis methods. | Primary outcome measure. Modified 0–13 scoring applied; critical domains summarized descriptively. |
| **PRISMA-2020** | Assesses completeness and transparency of reporting. | 27 reporting items across title, abstract, methods, search, selection, risk-of-bias, synthesis, and limitations. | Secondary outcome. Percentage adherence calculated; item-level coding retained. |
| **ROBIS** | Evaluates risk of bias in review conduct. | Four domains: eligibility criteria; identification/selection of studies; data collection/appraisal; synthesis and findings, plus overall risk-of-bias judgment. | Applied to reviews performing risk-of-bias assessment. |
| **AITDI (AI Transparency and Disclosure Index)** | Assesses quality and completeness of AI reporting in systematic reviews. | Six domains: tool identity; stage(s) of use; model version/date; prompting/configuration details; human-in-the-loop verification; data-governance/ethics statements. | Applied only to AI-assisted reviews as a secondary outcome; refinements undertaken in Aim 2. |

**Abbreviations:** AI: artificial intelligence; AITDI: AI Transparency and Disclosure Index; AMSTAR-2: A Measurement Tool to Assess Systematic Reviews version 2; PRISMA-2020: Preferred Reporting Items for Systematic Reviews and Meta-Analyses 2020; ROBIS: Risk of Bias in Systematic Reviews.

This appendix summarizes the primary and secondary outcomes and outlines the major domains and intended use of each appraisal tool applied in the study. Full item-level definitions can be found in the original AMSTAR-2, PRISMA-2020 and ROBIS development documents.

**References**:

1. Shea BJ, Reeves BC, Wells G, et al. AMSTAR 2: a critical appraisal tool for systematic reviews that include randomised or non-randomised studies of healthcare interventions, or both. *bmj*. 2017;358
2. Page MJ, McKenzie JE, Bossuyt PM, et al. The PRISMA 2020 statement: an updated guideline for reporting systematic reviews. *bmj*. 2021;372
3. Whiting P, Savović J, Higgins JP, et al. ROBIS: a new tool to assess risk of bias in systematic reviews was developed. *Journal of clinical epidemiology*. 2016;69:225-234.
