# Supplementary material for: Evaluating the Methodological Quality of Artificial Intelligence–Assisted Systematic Reviews: Protocol for a Mixed Methods Meta-Research Study
Source: JMIR Res Protoc. 2026 May 14;15:e90588. doi: 10.2196/90588 (PMC13175306; doi:10.2196/90588)
Supplement: Multimedia Appendix 2 [file resprot-v15-e90588-s002.docx]

**Multimedia Appendix 2.** Full search strategies for MEDLINE, Cochrane, and CINAHL (Executed December 3–5, 2025).

**Table 1.** MEDLINE (Ovid) Search Strategy — December 4, 2025

| **Line #** | **Terms** | **# of Results** |
| --- | --- | --- |
| **1** | Artificial Intelligence/ or Generative Artificial Intelligence/ or Machine Learning/ or Deep Learning/ or Natural Language Processing/ | 155490 |
| **2** | Large Language Models/ | 1431 |
| **3** | ("artificial intelligence" adj5 ("research question*" or search* or criteria or select* or evaluat* or synthesiz* or writing or write* or summariz* or screen* or duplicat* or analy*)).mp. | 8813 |
| **4** | (("AI" or "GenAI" or "generative artificial intelligence" or "machine learning" or "deep learning" or "natural language processing" or "large language model*" or LLM*) adj5 ("research question*" or search* or criteria or select* or evaluat* or synthesi#* or writing or write* or summari#* or screen* or duplicat* or analy*)).mp. | 56759 |
| **5** | (automat* adj3 (search* or extract* or select* or synthesi#* or interpret* or screen* or duplicat* or analy*)).mp. | 52361 |
| **6** | ("machine-assisted" adj5 ("research question*" or search* or criteria or select* or evaluat* or synthesiz* or writing or write* or summariz* or screen* or duplicat* or analy*)).mp. | 32 |
| **7** | (chatgpt* or OpenAI or GPT3 or GPT-3 or GPT4 or GPT-4 or gemini or claude or claude2 or "claude 2" or "microsoft copilot" or DeepSeek or mistral or mixtral or "BLOOM ai" or Llama or LLama2 or "perplexity ai" or distillerAI or "rayyan AI" or "elicit AI" or "research rabbit" or "undermind AI" or "litmaps" or "consensus AI" or "open evidence" or "Nvivo AI").mp. | 16204 |
| **8** | 1 or 2 or 3 or 4 or 5 or 6 or 7 | 247497 |
| **9** | (systematic review or meta-analysis).pt. | 400048 |
| **10** | meta-analysis/ or systematic review/ or systematic reviews as topic/ or meta-analysis as topic/ or "meta analysis (topic)"/ or "systematic review (topic)"/ or exp technology assessment, biomedical/ or network meta-analysis/ | 447355 |
| **11** | ((systematic* adj3 (review* or overview*)) or (methodologic* adj3 (review* or overview*))).ti,ab,kf. | 452417 |
| **12** | ((quantitative adj3 (review* or overview* or synthes*)) or (research adj3 (integrati* or overview*))).ti,ab,kf. | 21607 |
| **13** | ((integrative adj3 (review* or overview*)) or (collaborative adj3 (review* or overview*)) or (pool* adj3 analy*)).ti,ab,kf. | 49601 |
| **14** | (met analy* or metanaly* or technology assessment* or HTA or HTAs or technology overview* or technology appraisal*).ti,ab,kf. | 14855 |
| **15** | (meta-analy* or metaanaly* or systematic review* or biomedical technology assessment* or bio-medical technology assessment*).mp,hw. | 613602 |
| **16** | (cochrane or (health adj2 technology assessment) or evidence report).jw. | 23026 |
| **17** | (meta-analysis or systematic review).mp. | 573306 |
| **18** | (mixed adj3 treatment adj3 (meta-analy* or metaanaly*)).ti,ab,kf. | 189 |
| **19** | umbrella review*.ti,ab,kf. | 3261 |
| **20** | (multi* adj2 paramet* adj2 evidence adj2 synthesis).ti,ab,kf. | 16 |
| **21** | (multiparamet* adj2 evidence adj2 synthesis).ti,ab,kf. | 20 |
| **22** | (multi-paramet* adj2 evidence adj2 synthesis).ti,ab,kf. | 14 |
| **23** | 9 or 10 or 11 or 12 or 13 or 14 or 15 or 16 or 17 or 18 or 19 or 20 or 21 or 22 | 697048 |
| **24** | 8 and 23 | 7542 |
| **25** | limit 24 to yr="2023 -Current" | 5006 |

**Table 2.** Cochrane Library Search Strategy — December 3, 2025

| Line # | Terms | # of Results |
| --- | --- | --- |
| **1** | ("Artificial Intelligence" or "Generative Artificial Intelligence" or "Machine Learning" or "Deep Learning" or "Natural Language Processing").kw. | 3 |
| **2** | Large Language Models.kw. | 0 |
| **3** | ("artificial intelligence" adj5 ("research question*" or search* or criteria or select* or evaluat* or synthesiz* or writing or write* or summariz* or screen* or duplicat* or analy*)).af. | 24 |
| **4** | (("AI" or "GenAI" or "generative artificial intelligence" or "machine learning" or "deep learning" or "natural language processing" or "large language model*" or LLM*) adj5 ("research question*" or search* or criteria or select* or evaluat* or synthesi#* or writing or write* or summari#* or screen* or duplicat* or analy*)).af. | 132 |
| **5** | (automat* adj3 (search* or extract* or select* or synthesi#* or interpret* or screen* or duplicat* or analy*)).af. | 171 |
| **6** | ("machine-assisted" adj5 ("research question*" or search* or criteria or select* or evaluat* or synthesiz* or writing or write* or summariz* or screen* or duplicat* or analy*)).af. | 0 |
| **7** | (chatgpt* or OpenAI or GPT3 or GPT-3 or GPT4 or GPT-4 or gemini or claude or claude2 or "claude 2" or "microsoft copilot" or DeepSeek or mistral or mixtral or "BLOOM ai" or Llama or LLama2 or "perplexity ai" or distillerAI or "rayyan AI" or "elicit AI" or "research rabbit" or "undermind AI" or "litmaps" or "consensus AI" or "open evidence" or "Nvivo AI").af. | 113 |
| **8** | 1 or 2 or 3 or 4 or 5 or 6 or 7 | 400 |
| **9** | limit 8 to yr="2023 -Current" | 133 |

**Table 3.** CINAHL Search Strategy — December 5, 2025

| **#** | **Query** | **Results** |
| --- | --- | --- |
| **1** | (MH "Artificial Intelligence") OR (MH "Artificial Intelligence, Generative") OR (MH "Machine Learning") OR (MH "Deep Learning") OR (MH "Natural Language Processing") | 34,976 |
| **2** | XB (("artificial intelligence" N5 ("research question*" or search* or criteria or select* or evaluat* or synthesiz* or writing or write* or summariz* or screen* or duplicat* or analy*))) | 1,993 |
| **3** | XB ((("AI" or "GenAI" or "generative artificial intelligence" or "machine learning" or "deep learning" or "natural language processing" or "large language model*" or LLM*) N5 ("research question*" or search* or criteria or select* or evaluat* or synthesi#* or writing or write* or summari#* or screen* or duplicat* or analy*))) | 8,713 |
| **4** | XB ((automat* N3 (search* or extract* or select* or synthesi#* or interpret* or screen* or duplicat* or analy*))) | 7,084 |
| **5** | XB (("machine-assisted" N5 ("research question*" or search* or criteria or select* or evaluat* or synthesiz* or writing or write* or summariz* or screen* or duplicat* or analy*))) | 5 |
| **6** | XB ((chatgpt* or OpenAI or GPT3 or GPT-3 or GPT4 or GPT-4 or gemini or claude or claude2 or "claude 2" or "microsoft copilot" or DeepSeek or mistral or mixtral or "BLOOM ai" or Llama or LLama2 or "perplexity ai" or distillerAI or "rayyan AI" or "elicit AI" or "research rabbit" or "undermind AI" or "litmaps" or "consensus AI" or "open evidence" or "Nvivo AI")) | 3,125 |
| **7** | S1 OR S2 OR S3 OR S4 OR S5 OR S6 | 47,409 |
| **8** | (MH "meta analysis" OR MH "systematic review" OR MH "Technology, Medical/EV" OR PT "systematic review" OR PT "meta analysis" OR (((TI systematic* OR AB systematic*) N3 ((TI review* OR AB review*) OR (TI overview* OR AB overview*))) OR ((TI methodologic* OR AB methodologic*) N3 ((TI review* OR AB review*) OR (TI overview* OR AB overview*)))) OR (((TI quantitative OR AB quantitative) N3 ((TI review* OR AB review*) OR (TI overview* OR AB overview*) OR (TI synthes* OR AB synthes*))) OR ((TI research OR AB research) N3 ((TI integrati* OR AB integrati*) OR (TI overview* OR AB overview*)))) OR (((TI integrative OR AB integrative) N3 ((TI review* OR AB review*) OR (TI overview* OR AB overview*))) OR ((TI collaborative OR AB collaborative) N3 ((TI review* OR AB review*) OR (TI overview* OR AB overview*))) OR ((TI pool* OR AB pool*) N3 (TI analy* OR AB analy*))) OR ((TI "met analy*" OR AB "met analy*") OR (TI metanaly* OR AB metanaly*) OR (TI "technology assessment*" OR AB "technology assessment*") OR (TI HTA OR AB HTA) OR (TI HTAs OR AB HTAs) OR (TI "technology overview*" OR AB "technology overview*") OR (TI "technology appraisal*" OR AB "technology appraisal*")) OR (TI meta-analy* OR TI metaanaly* OR TI "systematic review*" OR TI "biomedical technology assessment*" OR TI "bio-medical technology assessment*" OR AB meta-analy* OR AB metaanaly* OR AB "systematic review*" OR AB "biomedical technology assessment*" OR AB "bio-medical technology assessment*" OR MW meta-analy* OR MW metaanaly* OR MW "systematic review*" OR MW "biomedical technology assessment*" OR MW "bio-medical technology assessment*") OR (SO Cochrane OR SO health technology assessment OR SO evidence report) OR (TI metaanaly* OR AB metaanaly*))) OR (TI "umbrella review*" OR AB "umbrella review*") OR ((TI multi* OR AB multi*) N2 (TI paramet* OR AB paramet*) N2 (TI evidence OR AB evidence) N2 (TI synthesis OR AB synthesis)) OR ((TI multiparamet* OR AB multiparamet*) N2 (TI evidence OR AB evidence) N2 (TI synthesis OR AB synthesis)) OR ((TI multi-paramet* OR AB multi-paramet*) N2 (TI evidence OR AB evidence) N2 (TI synthesis OR AB synthesis)) | 335,762 |
| **9** | "S7 AND S8  Limiters - Publication Date: 20230101-20260131" | 1,476 |

**Abbreviations:** AI = artificial intelligence; LLM = large language model; NLP = natural language processing; mp. = multi-purpose field search; af. = all fields; kw. = keyword; MH = CINAHL subject heading; N# = adjacency operator.
