# Supplementary material for: Evaluating the Methodological Quality of Artificial Intelligence–Assisted Systematic Reviews: Protocol for a Mixed Methods Meta-Research Study
Source: JMIR Res Protoc. 2026 May 14;15:e90588. doi: 10.2196/90588 (PMC13175306; doi:10.2196/90588)
Supplement: Multimedia Appendix 3 [file resprot-v15-e90588-s003.docx]

**Multimedia Appendix 3**. Data extraction variables and coding guidance.

| **Category** | **Variable** | **Description / Coding Guidance** |
| --- | --- | --- |
| **Bibliographic & Context** | Journal title | Extract as published. |
|  | Journal impact tier | Coded as Q1/Q2/Q3/Q4 (Scimago Journal Rank). |
|  | Country of corresponding author | Based on listed affiliation. |
|  | Author degree background | MD / PhD / MD-PhD / Other. |
|  | Review-group affiliation described (e.g., Cochrane, JBI, EPPI-Centre, Campbell, or other structured review organizations); specify group if applicable. | Yes / No |
|  | Publication timeline | Extract dates: received, accepted, published. |
|  | Funding source | Industry / Public / Mixed / Other. |
| **Review Characteristics** | Review question type | Intervention / Diagnostic accuracy / Prognostic / Etiology / Qualitative / Prevalence / Methodological / Other (specify). Code based on primary objective as stated in the SR. |
|  | Clinical domain | Based on major MeSH headings or topic classification. |
|  | Review type | Standard / Rapid / Living. |
|  | Included study designs | RCT only / RCT + observational / Mixed / Other. |
|  | Meta-analysis | Yes/No; if yes, extract model type and effect measure. |
|  | Search comprehensiveness | Number of databases searched. |
|  | Date of last search | Month and year. |
|  | Protocol registration | PROSPERO / OSF / Other / None. |
| **Extent of AI Use** | AI tools used | Tool/platform name(s) (e.g., ChatGPT, Rayyan, DistillerSR). |
|  | Stage of AI integration | Search / Screening / Data extraction / RoB assessment / Writing. |
|  | Model version or date | If reported (e.g., GPT-4, April 2024). |
|  | Human verification | Description of oversight; proportion of AI output checked. |
|  | Prompting detail | Yes/No; brief description if present. |
| **Process & Output Metrics** | Records retrieved, screened, included | Extract continuous counts. |
|  | Review team size | Number of authors. |
|  | Screening platform | Covidence / Rayyan / DistillerSR / Other. |
| **Timeliness & Impact** | Protocol-to-publication interval | Days (calculated from extracted dates). |
|  | Citation count | 12-month citation count (OpenAlex or Dimensions). |
|  | Altmetric attention score | Extract if reported. |

**Abbreviations:** AI: artificial intelligence; EPPI-Centre: Evidence for Policy and Practice Information and Co-ordinating Centre; JBI: Joanna Briggs Institute; MeSH: Medical Subject Headings; OSF: Open Science Framework; PROSPERO: International Prospective Register of Systematic Reviews; RCT: randomized controlled trial; RoB: risk of bias; SR: systematic review.
