# Supplementary material for: Evaluating the Methodological Quality of Artificial Intelligence–Assisted Systematic Reviews: Protocol for a Mixed Methods Meta-Research Study
Source: JMIR Res Protoc. 2026 May 14;15:e90588. doi: 10.2196/90588 (PMC13175306; doi:10.2196/90588)
Supplement: Multimedia Appendix 4 [file resprot-v15-e90588-s004.docx]

**Multimedia Appendix 4.** Semi-structured interview guide.

**Introduction and Orientation**

Thank you for agreeing to participate in this interview.
We are exploring perspectives on the use of artificial intelligence (AI) in systematic reviews (SRs). We are interested in your experiences, views, and expectations regarding rigor, transparency, reproducibility, and trust in AI-assisted evidence synthesis. There are no right or wrong answers. You may skip any question. With your permission, this interview will be audio-recorded for transcription.

**Section 1. Background and Experience**

1. Can you tell me about your role and your experience with SRs?
   Probes:
   - Have you authored, reviewed, used, or overseen SRs?
   - In what capacity do SRs influence your work?
2. What experience, if any, do you have with AI or machine learning tools in research or clinical contexts?
   Probes:
   - Screening tools (e.g., Rayyan classifier)
   - LLMs (e.g., ChatGPT, Claude)
   - Automation tools in SR software

**Section 2. Perceptions of AI-Assisted SRs**

1. What comes to mind when you think about AI being used in SR workflows?
   Probes:
   - Initial reactions: optimism, skepticism, concerns
   - Perceived role of AI in supporting or replacing human judgment
2. In your view, what are the potential benefits of using AI in SRs?
   Probes:
   - Efficiency or time savings
   - Reducing workload
   - Consistency or reproducibility
3. What are the potential risks or downsides?
   Probes:
   - Accuracy concerns
   - Hidden errors
   - Over-reliance on AI
   - Erosion of human expertise

**Section 3. Standards for Rigor, Transparency, and Oversight**

1. When evaluating an SR, what signals or criteria tell you that it is rigorous and trustworthy?
   Probes:
   - Methodological standards
   - Transparency of workflow
   - Disclosure practices
2. How important is transparency about how AI was used in a SR?
   Probes:
   - What information should be reported?
   - Are technical details (e.g., prompts, model versions) necessary?
   - Minimum vs. ideal reporting standards
3. What level of human oversight do you believe is necessary when AI tools are used?
   Probes:
   - Which tasks require human judgment
   - Acceptable/unsafe tasks for AI
   - Accountability for errors
4. Are there “red lines”: situations where you believe AI should not be used in SRs?
   Probes:
   - Risk-of-bias appraisal
   - Interpretation or conclusion writing
   - Combining AI-generated steps without verification

**Section 4. Expectations for Disclosure and Governance**

1. What information should journals, reviewers, or guideline developers require authors to disclose about AI use?
   Probes:
   - Tool identity, version
   - Stage of use
   - Prompts / parameters
   - Human verification steps
   - Data privacy/ethics considerations
2. In your view, who is responsible for ensuring the accuracy and integrity of AI-assisted SRs?
   Probes:
   - Authors, editors, peer reviewers
   - Tool developers
   - Journals or institutions

**Section 5. Acceptability and Future Use**

1. Under what conditions would you feel comfortable using an AI-assisted SR to inform clinical practice, guideline decisions, or policy?
   Probes:
   - Threshold conditions
   - Safeguards needed
   - Transparency requirements
2. Do you foresee AI changing the role of SRs in the future?
   Probes:
   - New skills required
   - Redistribution of tasks
   - Impact on training
3. How, if at all, do you think increasing use of AI in SR workflows should influence how SRs are evaluated or valued in academic settings (e.g., promotion or funding decisions)?
4. Do you think the role of SRs in the evidence hierarchy is changing in the context of increasing AI use? If so, how?

**Section 6. Reflection and Closing**

1. Is there anything else you would like to share about AI in SR that we have not discussed?
2. Do you have any recommendations for researchers, journals, or guidelines groups as AI becomes more integrated into SR workflows?

**End of Interview**

Thank you very much for your time and insights. Your perspectives will help inform guidance on transparency, oversight, and best practices for AI-assisted systematic reviews.
